# Evaluating Retrieval-Augmented Generation-Large Language Models for Infective Endocarditis Prophylaxis: Clinical Accuracy and Efficiency

**DOI:** 10.1016/j.identj.2025.109344

**Published:** 2025-12-25

**Authors:** Paak Rewthamrongsris, Vivat Thongchotchat, Jirayu Burapacheep, Vorapat Trachoo, Zohaib Khurshid, Thantrira Porntaveetus

**Affiliations:** a Department of Anatomy, Faculty of Dentistry, Center of Artificial Intelligence and Innovation (CAII), Center of Excellence for Dental Stem Cell Biology, Chulalongkorn University, Bangkok, Thailand; b Center of Excellence in Precision Medicine and Digital Health, Department of Physiology, Faculty of Dentistry, Chulalongkorn University, Bangkok, Thailand; c Stanford University, Stanford, California, USA; d Department of Oral and Maxillofacial Surgery, Faculty of Dentistry, Chulalongkorn University, Bangkok, Thailand; e Department of Prosthodontics and Dental Implantology, College of Dentistry, King Faisal University, Al-Ahsa, Kingdom of Saudi Arabia; f FutureDent Digital Center, Chulalongkorn University Implant and Esthetic Center (CUIE), Faculty of Dentistry, Chulalongkorn University, Bangkok, Thailand; g Clinic of General-, Special Care and Geriatric Dentistry, Center for Dental Medicine, University of Zurich, Zurich, Switzerland

**Keywords:** Artificial intelligence, Chatbots, Clinical Decision support systems, Health care access, Large language models, Retrieval augmented generation

## Abstract

**Introduction and Aims:**

The use of large language models (LLMs) in healthcare is expanding. Retrieval-augmented generation (RAG) addresses key LLM limitations by grounding responses in domain-specific, up-to-date information. This study evaluated RAG-augmented LLMs for infective endocarditis (IE) prophylaxis in dental procedures, comparing their performance with non-RAG models assessed in our previous publication using the same question set. A pilot study also explored the utility of an LLM as a clinical decision support tool.

**Methods:**

An established IE prophylaxis question set from previous research was used to ensure comparability. Ten LLMs integrated with RAG were tested using MiniLM L6 v2 embeddings and FAISS to retrieve relevant content from the 2021 American Heart Association IE guideline. Models were evaluated across five independent runs, with and without a preprompt (‘You are an experienced dentist’), a prompt-engineering technique used in previous research to improve LLMs accuracy. Three RAG-LLMs were compared to their native (non-RAG) counterparts benchmarked in the previous study. In the pilot study, 10 dental students (5 undergraduate, 5 postgraduate in oral and maxillofacial surgery) completed the questionnaire unaided, then again with assistance from the best performing LLM. Accuracy and task time were measured.

**Results:**

DeepSeek Reasoner achieved the highest mean accuracy (83.6%) without preprompting, while Grok 3 beta reached 90.0% with preprompting. The lowest accuracy was observed for Claude 3.7 Sonnet, at 42.1% without preprompts and 47.1% with preprompts. Preprompting improved performance across all LLMs. RAG’s impact on accuracy varied by model. Claude 3.7 Sonnet showed the highest response consistency without preprompting; with preprompting, Claude 3.5 Sonnet and DeepSeek Reasoner matched its performance. DeepSeek Reasoner also had the slowest response time. In the pilot study, LLM support slightly improved postgraduate accuracy, slightly reduced undergraduate accuracy, and significantly increased task time for both.

**Conclusion:**

While RAG and prompting enhance LLM performance, real-world utility in education remains limited.

**Clinical relevance:**

LLMs with RAG provide rapid and accessible support for clinical decision-making. Nonetheless, their outputs are not always accurate and may not fully reflect evolving medical and dental knowledge. It is crucial that clinicians and students approach these tools with digital literacy and caution, ensuring that professional judgment remains central.

## Introduction

Large language models (LLMs) are a subset of generative artificial intelligence (AI) that can produce human-like text by leveraging advanced natural language processing techniques developed through large-scale deep-learning training.^1^^,^^2^ The rapid development of LLMs lead to many applications in medical and dental field to facilitate patient care, enhance clinical decision making, streamline clinical workflow.^3^^,^^4^

Retrieval-augmented generation (RAG) has emerged as a promising method to mitigate hallucinations and outdated knowledge in LLM outputs. By integrating a retrieval module that supplies relevant external documents (eg, clinical guidelines, research articles), RAG aims to ground model responses in up-to-date, domain-specific evidence.^5^^,^^6^ This capability is especially crucial in medical and dental domains, where new research findings emerge continuously and any misinformation can have serious implications for patient care and scientific validity.^7^ In a typical RAG pipeline, the system first fetches pertinent documents or data from a domain-specific database or literature based on the user’s query, then incorporates this retrieved content into the LLM’s input context to inform the subsequent generated response.^6^ RAG generally improves the factual accuracy and specificity of the model’s output while mitigating the risk of hallucinations. Recent studies have shown that RAG-augmented chatbots enhance the performance of language models in medical^8^, ^9^, ^10^ and dental^11^ fields, enabling more accurate and evidence-based responses.

Infective endocarditis (IE) is a life-threatening condition, and dental procedures can increase the risk by allowing bacteria to enter the bloodstream, especially in individuals with certain conditions. To minimize this risk, antibiotic prophylaxis is recommended for high-risk patients.^12^^,^^13^ However, these guidelines are not static and may evolve as new evidence becomes available, for instance, the 2007 American Heart Association (AHA) guideline for antibiotic prophylaxis against IE in dental procedures recommendations was updated to 2021 version to reflect emerging research and clinical insights.^12^^,^^13^ Our previous study found that GPT-4o, when guided by prompt engineering, achieved the highest accuracy (80%) in answering questions based on the 2021 AHA guideline.^14^ However, the model still cited outdated information from the 2007 guidelines and showed limitations as a standalone clinical tool.

In this study, we aimed to assess the effects of integrating RAG, using the 2021 AHA guideline as the data source, on the performance of LLMs. Additionally, we conducted a pilot study to evaluate the assistive capabilities of the LLMs in clinical scenarios by comparing the performance scores of five undergraduate and five postgraduate oral and maxillofacial surgery (OMFS) dental students with and without access to LLMs, on the same set of questions. We hypothesized that RAG-implemented LLMs would demonstrate high accuracy in answering clinical queries and would outperform their non-RAG (native) counterparts. In addition, regarding the assistive capabilities of LLMs for dental students, we hypothesized that the use of LLM assistance would enhance students’ performance. This study not only seeks to improve the accuracy and relevance of LLM-generated responses in clinical contexts but also underscores the potential of RAG to advance dental research and clinical practice.

## Material and methods

### Dataset construction

We used the same 28 true/false questions previously employed to evaluate LLMs’ performance, allowing us to directly compare the performance of LLMs with access to RAG in this study against LLMs’ performance without RAG in the previous study.^14^ The questions were developed based on the 2021 AHA scientific statement on the IE prevention.^13^ Each question and its corresponding reference answer were reviewed and validated by an oral and maxillofacial surgeon holding dual degrees in medicine and dentistry. The question set addressed key elements of IE prophylaxis related to dental procedures, encompassing indications, contraindications, antibiotic selection, dosage, and timing. The chosen sample size was consistent with prior research assessing the performance of LLMs in healthcare applications.^15^^,^^16^ The checklist for the study of AI in dental research has been reviewed (Supplementary Table S1).^17^

### RAG implementation

The RAG implementation, question querying, and response parsing were conducted by a data scientist holding a PhD and with 5 years of professional experience. The process began with the ingestion and parsing of the AHA Guidelines PDF, converting it into machine-readable text while preserving the document’s logical structure. We used a rule-based segmentation method to identify major sections by detecting typographic features, followed by chunking each section into overlapping text segments (approximately 1000 characters with 200-character overlaps). This ensured contextual continuity across segment boundaries, resulting in 117 semantically coherent chunks. Each segment was embedded into vector space using the MiniLM-L6-v2, a lightweight, high-performance model fine-tuned for semantic similarity tasks. MiniLM-L6-v2 captures contextual relationships between words and phrases, enabling the system to understand meaning beyond surface-level keywords. These embeddings provide a numerical representation of each text segment’s semantic content. These embeddings were then indexed using FAISS, an open-source library optimized for efficient vector similarity search and clustering at scale. FAISS allows rapid retrieval of the most relevant text segments by comparing the cosine similarity between the embedded question and the stored document vectors. This ensures that semantically related content is retrieved even when exact keyword matches are absent.

At query time, questions were embedded in the same vector space and matched to the most relevant guideline segments. These retrieved passages were then provided as context to the language model to generate answers grounded in the AHA recommendations.

### Model selection and querying

We evaluated 10 LLMs developed by six companies, both open-source and proprietary. The seven most recent models at the time were: GPT-4.5 Preview, GPT-o3-mini from OpenAI, Claude 3.7 Sonnet from Anthropic, Gemini 2.0 Pro from Google DeepMind, DeepSeek Reasoner, Llama 3 70b from Meta, and Grok 3 beta from xAI.

All questions were queries via respective Application Programming Interface (API) endpoints to ensure no memory of previously questions and standardized process. All parameters of the LLMs were left at their default settings according to each company’s configuration. The same preprompt (‘You are an experienced dentist.’) and no-preprompt strategy were used to compare to previous published report. All experiments were conducted 5 times independently between 20 March and 2 April 2025. To specifically assess the internal consistency of responses and isolate performance from temporal factors, these five repetitions were executed consecutively for each model. The 2-week total timeframe was deemed sufficiently short to mitigate the risk of confounding variables, such as major model updates.

### Comparing RAG and non-RAG LLMs

In a previous study, the accuracy of LLMs without RAG implementation was reported.^14^ To directly compare the effect of RAG on LLM accuracy, we additionally tested three legacy models corresponding to those benchmarked in the previous study, which were the top performers from each developer: GPT-4o (OpenAI), Claude 3.5 Sonnet (Anthropic), and Gemini 1.5 Pro (Google DeepMind). The results of these RAG-implemented models were then compared with the previously reported benchmark values.

### Response parsing

The raw text output from the LLMs for each true/false question was systematically parsed and scored. First, each response was classified as either ‘true’ or ‘false’ based on its content. Responses containing affirmative language (eg, ‘true’, ‘generally true’, ‘partially true’) were categorized as ‘true’. Conversely, responses with negative language (eg, ‘false’ or other forms of negation) were categorized as ‘false’.

Second, to determine accuracy, this classified answer was compared against the predetermined correct answer from the AHA guidelines. A response was marked as correct only if its classification matched the ground truth. Responses that did not match, or those that expressed uncertainty or a refusal to answer, were marked as incorrect. This methodology is consistent with our prior work.^14^

### Participant recruitment

This study was approved by the Human Research Ethics Committee, Faculty of Dentistry, Chulalongkorn University (Approval No. 109/2024). To assess whether LLM could enhance the clinical decision-making abilities of dental professionals, five sixth-year (final-year) consented dental students and five consented dentists enrolled in a postgraduate OMFS program were recruited using convenience and snowball sampling methods. To ensure consistency with the experimental design used for models evaluation, a sample size of five participants was selected, corresponding to the five independent runs conducted during the LLMs performance assessment.

Each participant was instructed to complete the same questionnaire via online platform twice, answering as accurately as possible. In the first round, participants completed the questionnaire without access to any tools or external resources. After at least 1-month interval, they were asked to complete the same questionnaire again, this time with assistance from the best-performing chatbot identified in this study. The time taken to complete the questionnaire was recorded in both rounds. To reflect natural use of LLMs in clinical settings, participants were not instructed to use prompt engineering or any specific method.

### Statistical analysis

LLMs responses were evaluated against predetermined ground answers and classified as either correct or incorrect. Statistical analyses were conducted using Prism 10 (version 10.1.0; GraphPad Software). Mean accuracy across all runs was calculated in the same way as in our previous study.^14^

Intermodel comparisons were performed using the Kruskal–Wallis test, followed by Dunn’s multiple comparisons test. The Wilcoxon matched-pairs signed-rank test was used to compare performance between preprompted and nonpreprompted strategies, LLMs with and without RAG, and participants’ scores and time taken to complete the questionnaire with and without LLM assistance.

Model consistency was defined as the percentage of questions for which an LLMs produced identical responses across all five runs, regardless of correctness. Statistical significance was set at *P* < .05.

## Results

### No-preprompt condition

**Fig. 1:**
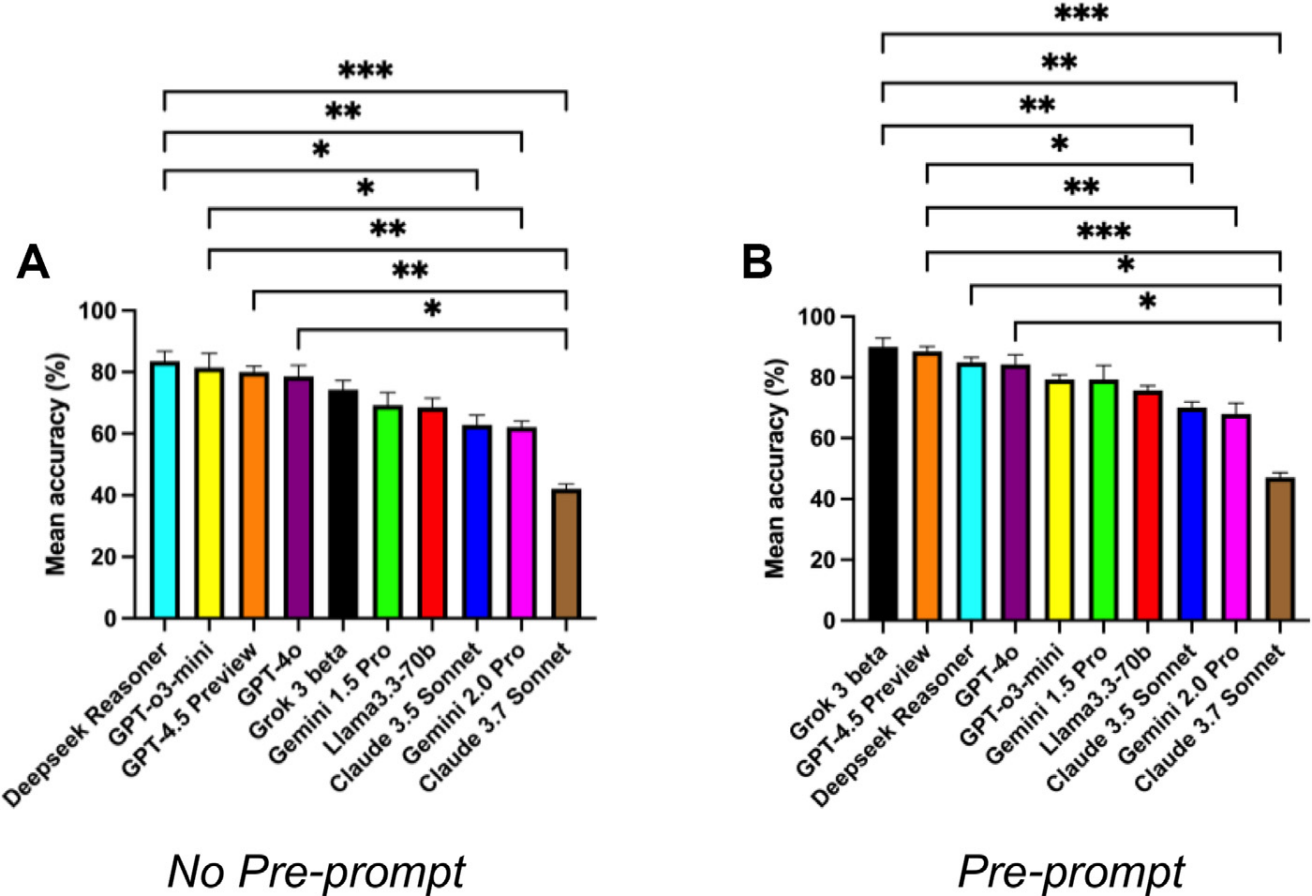
Mean accuracy of 10 LLMs tested. (A) Without a preprompt, and (B) with a preprompt strategy. Asterisks indicate statistically significant differences between models. *P < .05, **P < .01, ***P < .001.

DeepSeek Reasoner attained the highest mean accuracy (83.6% ± 3.2), surpassing all comparator models (Figure 1A). The next three positions were occupied by the ChatGPT family: GPT-o3-mini, GPT-4.5 Preview, and GPT-4o, respectively. Performance then declined sequentially for Grok 3 beta, Gemini 1.5 Pro, Llama 3 70b, Claude 3.5 Sonnet, and Gemini 2.0 Pro. Claude 3.7 Sonnet exhibited the lowest mean accuracy (42.1% ± 1.6). Pairwise differences that reached statistical significance are shown in Figure 1A. Comprehensive descriptive statistics are provided in Table.

**Table:** The accuracy, performance, consistency, and response time of LLMs without and with preprompts.

	Mean	Std. deviation	Lower 95% CI of mean	Upper 95% CI of mean	Response time per query (s)	Consistency (%)	Mean	Std. deviation	Lower 95% CI of mean	Upper 95% CI of mean	Response time per query (s)	Consistency (%)
	Without preprompts						With preprompts					
Claude 3.5 Sonnet	62.9	3.2	58.9	66.8	4.1	92.9	70.0	2.0	67.6	72.4	4.1	96.4
Claude 3.7 Sonnet	42.1	1.6	40.2	44.1	3.7	96.4	47.1	1.6	45.7	49.1	4.0	96.4
Deepseek Reasoner	83.6	3.2	79.6	87.5	42.7	89.3	85.0	1.6	83.0	87.0	37.8	96.4
Gemini 1.5 Pro	69.3	4.1	64.2	74.3	2.0	85.7	79.3	4.7	73.5	85.1	2.2	78.6
Gemini 2.0 Pro	62.1	2.0	59.7	64.6	9.1	82.1	67.9	3.6	63.4	72.3	9.7	82.1
GPT-4.5 Preview	80.0	2.0	77.6	82.4	10.1	89.3	88.6	1.6	86.6	90.6	11.5	89.3
GPT-4o	78.6	3.6	74.1	83.0	2.1	89.3	84.3	3.2	80.3	88.3	2.3	89.3
Llama3.3-70b	68.6	3.0	64.9	72.3	2.6	92.9	75.7	1.6	73.7	77.7	2.1	89.3
GPT-o3-mini	81.4	4.7	75.7	87.2	5.5	85.7	79.3	1.6	77.3	81.3	6.1	89.3
Grok 3 beta	74.3	2.9	70.6	78.0	2.9	89.3	90.0	3.0	86.3	93.7	3.1	85.7

### Preprompt condition

**Fig. 2:**
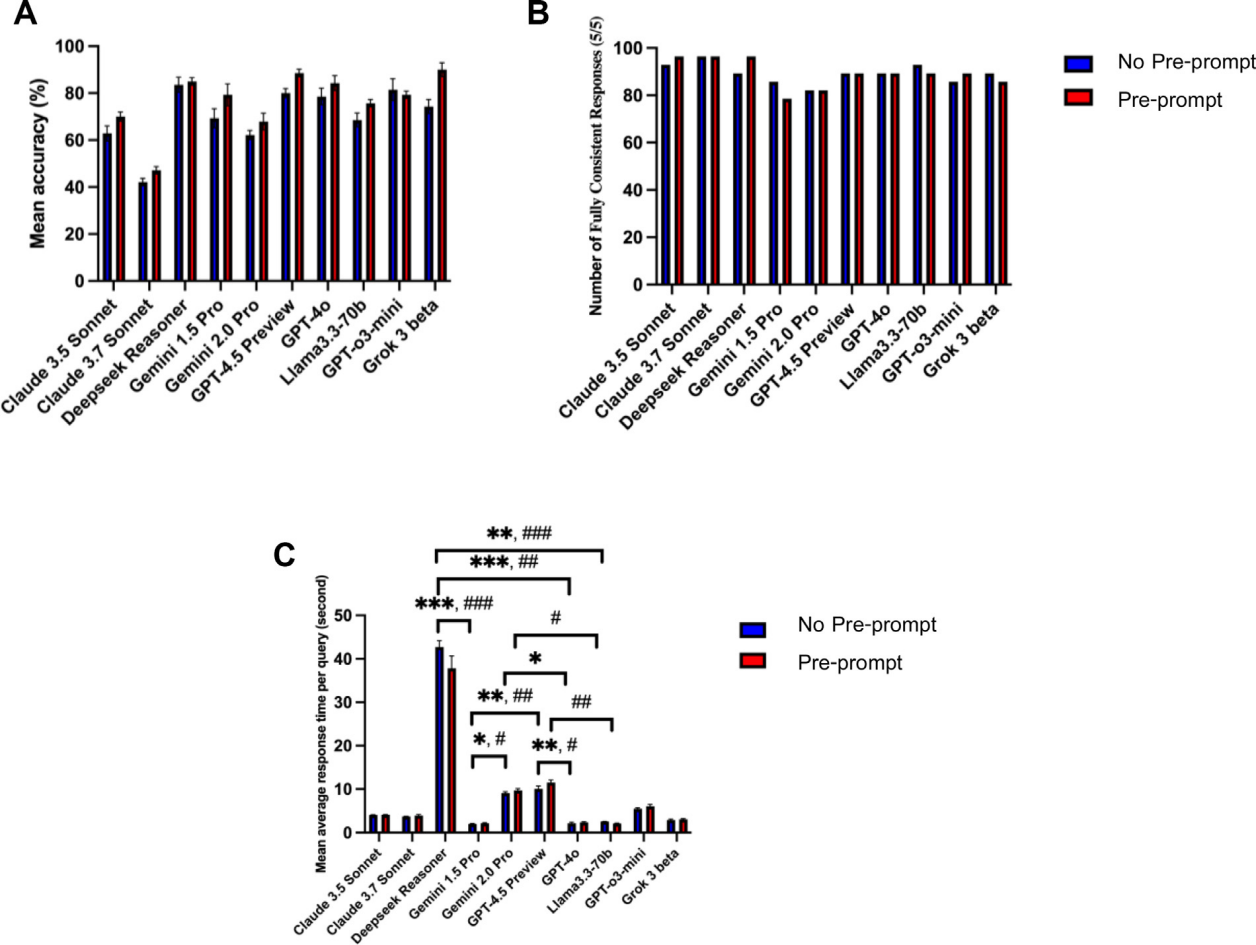
Comparison of the effects of no-preprompt and preprompt strategies on LLMs. (A) Mean average accuracy across LLMs. (B) Percentage of fully consistent responses over five runs. (C) Mean response time per query for each LLM. Asterisks indicate statistically significant differences between models without a preprompt, and sharp signs (#) indicate statistically significant differences between models with a preprompt. *,#*P* < .05, **,##*P* < .01, ***,###*P* < .001.

Preprompting increased the mean accuracy of all models, though not significantly (Figure 2A). Grok 3 beta became the top performer (90.0% ± 3.0), while DeepSeek Reasoner dropped to third. The three ChatGPT models remained in the leading cluster. Gemini 1.5 Pro and GPT-o3-mini had identical mean accuracies (79.3%), but Gemini 1.5 Pro showed greater variability. The five lowest-ranking models maintained their relative order with improved accuracy. Significant differences are shown in Figure 1B; full descriptive statistics are in Table.

### Consistency

Under the no-preprompt condition, Claude 3.7 Sonnet showed the highest response consistency at 96.4%. With preprompting, Claude 3.7 Sonnet, Claude 3.5 Sonnet, and DeepSeek Reasoner all achieved 96.4%. Consistency increased for Claude 3.5 Sonnet, DeepSeek Reasoner, and GPT-o3-mini, but decreased for Gemini 1.5 Pro, Llama 3.3 70b, and Grok 3 beta. Claude 3.7 Sonnet, Gemini 2.0 Pro, GPT-4.5 Preview, and GPT-4o showed no change (Figure 2B).

### Response time

Response time of LLMs showed the same pattern in both no-preprompt and preprompt (Figure 2C). Deepseek Reasoner exhibited the longest response time, averaging 42.7 seconds per query without a preprompt, followed by GPT-4.5 Preview and Gemini 2.0 Pro, respectively. Preprompt strategy increased response time of most LLMs except for Deepseek Reasoner and Llama 3.3 70b. Response time differences between no-preprompt and preprompt of all LLMs were not statistically significant.

### Effect of RAG on model accuracy

**Fig. 3:**
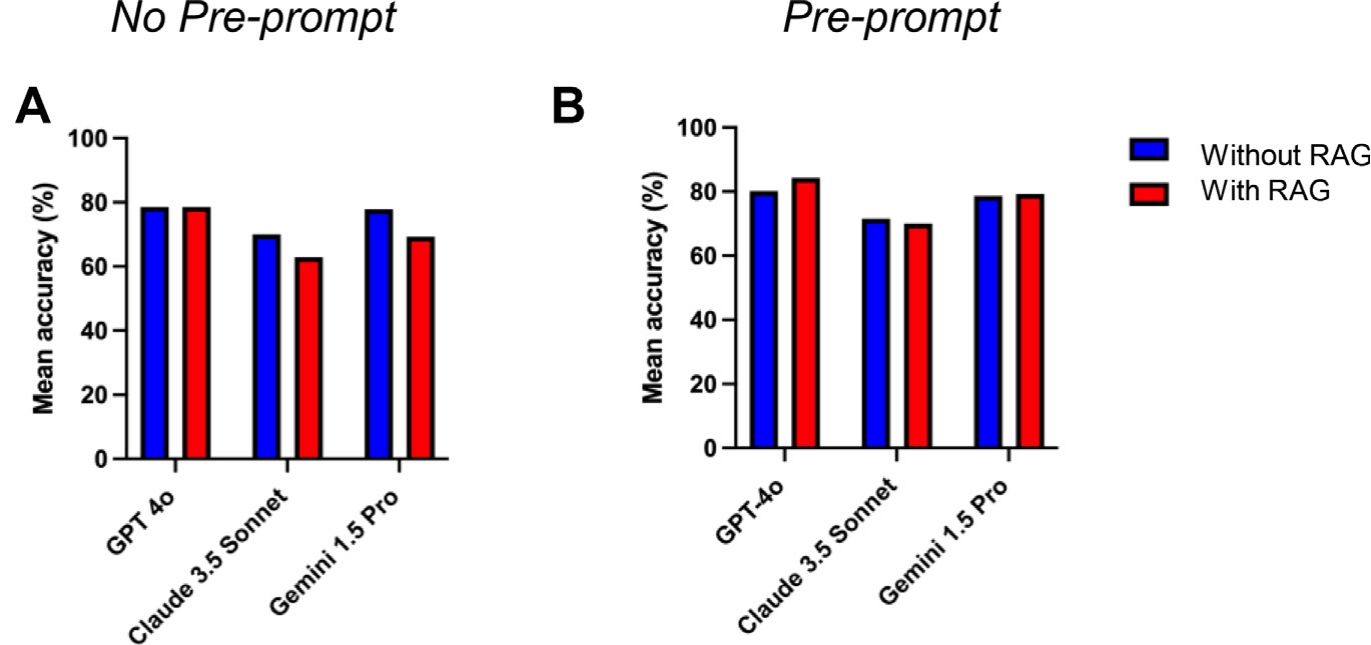
Comparison of mean accuracy between three LLMs with and without RAG, based on a previously published study. (A) Without preprompt. (B) With preprompt. Asterisks indicate statistically significant differences between models. *P < .05, **P < .01, ***P < .001.

In the absence of a preprompt (Figure 3A), GPT-4o with RAG matched its non-RAG baseline mean accuracy (78.6%) while exhibiting reduced standard deviation (SD = 3.6 with RAG and 5.1 without RAG). Conversely, RAG resulted in statistically nonsignificant decreases in mean accuracy for both Claude 3.5 Sonnet and Gemini 1.5 Pro. With the introduction of a preprompt (Figure 3B), RAG produced nonsignificant accuracy gains for GPT-4o and Gemini 1.5 Pro, but a nonsignificant decline for Claude 3.5 Sonnet.

### LLMs assisting undergraduate and postgraduate dental students

**Fig. 4:**
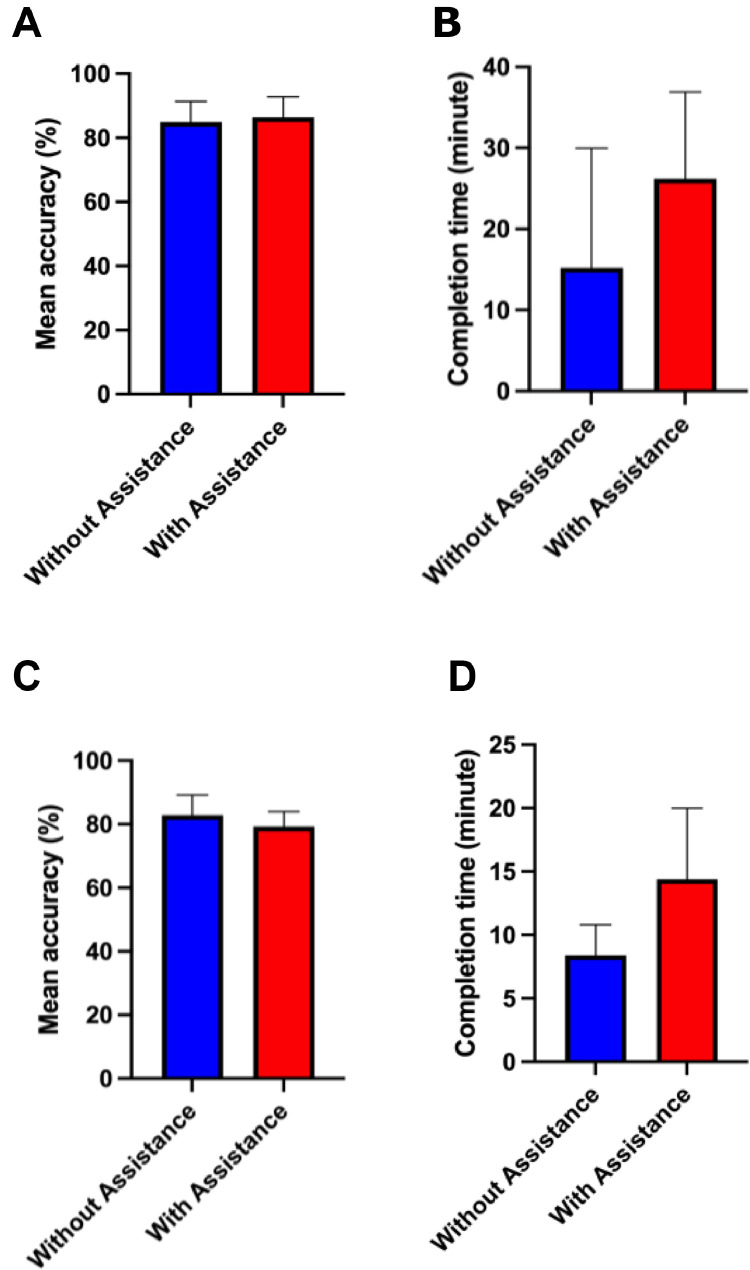
Comparison of dental practitioners’ performance on a question set with and without LLM assistance. (A) Mean accuracy of postgraduate students enrolled in an oral and maxillofacial surgery program. (B) Mean time taken to complete the question set by postgraduate students. (C) Mean accuracy of sixth-year undergraduate dental students. (D) Mean time taken to complete the question set by undergraduate students. Asterisks indicate statistically significant differences between models. *P < .05, **P < .01, ***P < .001.

DeepSeek Reasoner was chosen as the assisting tool for this phase due to its highest mean accuracy without a preprompt, reflecting the strongest raw performance. There were no participant dropouts. Among postgraduate students specializing in OMFS, overall scores showed a nonsignificant improvement (85.0% ± 6.4 to 86.4% ± 6.4 mean accuracy) with LLM assistance, accompanied by longer completion times from 15.2 to 26.2 minutes (Figure 4A,B). Specifically, Three participants improved their scores, two declined, and four of five took more time. For undergraduate students, four out of five participants showed a decrease in their scores during the second round of the questionnaire, resulting in a nonsignificant drop from 82.9% ± 6.4 to 79.3% ± 4.67. All five undergraduates spent more time with LLM assistance, increasing average completion time from 8.4 to 14.4 minutes (Figure 4A,B).

## Discussion

Among the 10 LLMs evaluated, the top five performing models, three from the ChatGPT family, DeepSeek Reasoner, and Grok 3 beta, consistently ranked highest across both preprompt and no-preprompt conditions when responding to questions specifically related IE prophylaxis based on the 2021 AHA guideline. The comparative analysis between these conditions corroborated findings from our previous work,^14^ indicating that preprompting can enhance LLM performance. Superior performance was observed with open-source models: Grok 3 beta, with preprompting, achieved 90.0% ± 3.0 mean accuracy, and DeepSeek, without preprompting, achieved 83.6%. ± 3.2 These accuracies exceed previously established benchmarks, including the 80.0% ± 0.84 mean accuracy of the top performer, GPT-4o (without RAG), reported in our prior study, and surpass the performance of GPT-4o with RAG in the current study. The results of our study did not fully support our stated hypotheses. First, the hypothesis that RAG implementation would lead to high accuracy was challenged by the significant performance variability observed among the tested models. Second, the hypotheses predicting a performance gain for LLMs using RAG and an enhancement in student performance were also not supported, as neither of these effects reached statistical significance.

The current findings indicate a potential for open-source models to achieve superior accuracy over proprietary models through more effective utilization of RAG. Regarding specific task performance, all evaluated LLMs, under both preprompt and no-preprompt conditions, accurately identified the 2021 AHA guideline as the most current version, superseding the 2007 iteration. This contrasts with an observation from our prior research, where, unexpectedly, three LLMs augmented with RAG failed to outperform their native (non-RAG) versions. In comparative human performance, both postgraduate and undergraduate dental practitioners achieved scores analogous to the top three LLMs when the models operated without preprompting. However, when benchmarked against preprompted LLMs, practitioner scores were positioned between those of the fourth- and fifth-ranked models.

RAG has been widely reported to enhance the performance of LLMs. For instance, a study in neurology demonstrated that a document-augmented GPT-4 achieved 87% accuracy, a significant improvement from 60% when operating without retrieval.^18^ Similarly, ‘PerioGPT’, a dental chatbot integrating GPT-4 with a curated periodontal knowledge base, attained an accuracy of 81% to 87%, outperforming both general-purpose GPT-4 and other existing dental AI tools.^19^ Furthermore, a systematic review concluded that RAG improved clinical question-answering efficacy by a factor of 1.35 relative to base models, although this enhancement exhibited considerable variability across different clinical domains.^20^

However, RAG does not invariably enhance LLM performance and may introduce novel challenges. For instance, one study reported that integrating even high-quality content from Harrison’s Principles of Internal Medicine led to LLM accuracy changes ranging from –10.3% to +1.9%; notably, GPT-4o exhibited less performance degradation than Claude 3.5 Sonnet and the Gemma family models (the open-source counterparts to Gemini).^21^ Corroborating this trend of differential model susceptibility, our current investigation also observed diminished performance in Claude 3.5 Sonnet and Gemini 1.5 Pro post-RAG augmentation, while GPT-4o demonstrated a marginal improvement. Furthermore, a study assessing the performance of LLMs in neurology reported that while incorporating RAG substantially enhanced GPT-4o’s accuracy, the LLaMA model augmented with online RAG remained prone to errors that were highly dependent on the quality and relevance of the information retrieved, underscoring its vulnerability to inconsistent or low-quality sources.^18^

Recent evaluations underscore that the efficacy of LLMs employing RAG is highly contingent upon the quality of the retrieved documents. For instance, models such as Phi 3.5 and Qwen 2.5 demonstrated discernible improvements in both accuracy and confidence calibration when supplied with relevant contextual information. Conversely, Llama 3.1 and Gemma 2 exhibited minimal performance gains, even with access to relevant documents and were more susceptible to being misguided by irrelevant information. The same study also elucidated the ‘Lost in the Middle’ phenomenon, wherein the placement of pertinent content amidst less critical text (eg, following answer choices) can degrade performance; accuracy was generally superior when documents were prepended or appended to the question.^22^ Notably, Llama 3.1 70b consistently underperformed relative to its non-RAG baseline across all tested RAG conditions.^22^

The present observations corroborate those of Maekawa et al, who reported that smaller and medium-sized models, including Flan T5 and Mistral 7B, derive significant benefit from RAG owing to their inherently limited baseline factual recall. Conversely, larger models such as Llama 2 13B, GPT-3.5, and GPT-4 frequently exhibit minimal or even negative performance changes with RAG on common questions, presumably because their extensive internal knowledge diminishes the utility of external information retrieval.^23^ A consonant pattern was observed in a domain-specific ophthalmology study, wherein RAG substantially improved the performance of Baichuan 13B, a medium-sized open-source language model. Nevertheless, the RAG-enhanced Baichuan 13B achieved performance merely comparable to that of the native GPT-4o, a larger proprietary model already demonstrating high concordance with expert consensus.^24^ Similarly, our study posits that the 2021 AHA guideline represents a widely known fact likely incorporated within the pretraining datasets of large models, consequently rendering RAG of limited value or even detrimental to their performance. Collectively, these results support the hypothesis that while RAG can substantially augment the capabilities of smaller and medium-sized models, its utility decreases and may become counterproductive for state-of-the-art models possessing robust internal factual recall.

The suboptimal performance of RAG can also stem from a semantic gap between user queries and clinical literature, especially in medical and dental fields, where vocabulary differences reduce retrieval precision and risk, including irrelevant or outdated content. This is particularly true for case-based questions, whose phrasing often differs from source documents, leading to poorer RAG outcomes than knowledge-inquiring questions.^18^ LLM possess finite attention capacities and may fail to identify crucial details embedded within voluminous or inadequately structured retrieved documents, consequently degrading response quality.^21^ While proprietary models may gain less from RAG due to high baselines, open-source models, though benefiting more, are also more prone to errors from suboptimal retrieval.^25^ The structural characteristics of source documents also modulate RAG performance. Ke et al posited that international guidelines, which amalgamate explanatory text with tables and figures, yield superior outcomes compared to local guidelines predominantly reliant on tabular or visual formats. This observation aligns with our findings, as the 2021 AHA guideline primarily presents critical information, such as antibiotic regimens and prophylaxis indications, in tabular formats, potentially impeding effective information retrieval.^25^

Congruent with these mixed outcomes, our study showed that DeepSeek Reasoner, despite achieving the highest mean accuracy among LLMs without preprompting, provided negligible benefit to participants’ overall task performance. This trend is consistent with findings from Glick et al^26^ and parallels observations by Devlin et al,^27^ where AI interventions did not substantially improve outcomes for users with less experience. In our investigation, DeepSeek Reasoner failed to augment the performance of undergraduate students (characterized by less experience), whereas postgraduate students demonstrated only a slight increase in mean scores. The prolonged questionnaire completion time is a key finding directly linked to the selection of the DeepSeek Reasoner. Its average response time of approximately 40 seconds per query resulted in a cumulative waiting period of nearly 7 minutes for students, highlighting a critical tradeoff between accuracy and efficiency. This delay presents a significant barrier to practical application in time-sensitive clinical or educational environments. We now emphasize that future evaluations must consider response time as a coprimary metric alongside accuracy to assess the real-world viability of LLMs. A limitation to consider is the differing timeframes in which the RAG evaluation and the native performance benchmark (from our prior study) were conducted. While this introduces the possibility of intervening model updates influencing results, the observed lower accuracy in the later RAG assessment indicates that such updates likely did not confer material performance improvements in the evaluated medical and dental contexts. Another acknowledged limitation is the modest sample size of five undergraduate and five postgraduate participants. This specific cohort size was intentionally selected to align with the five independent LLM evaluation runs, a design choice made to conceptualize each LLM run as equivalent to one human participant, thus enabling a more direct and interpretable comparative analysis. While the aggregated data are informative, there was notable interindividual variability in student performance. These differences may stem from unmeasured factors such as baseline knowledge, digital literacy, and student concentration. This highlights that the utility of an LLM is not uniform and underscores the importance of considering human factors when developing personalized AI tutors and promoting equitable learning outcomes. Finally, our study did not include a human-control equivalent of the RAG process. Future work would be strengthened by comparing the performance of LLM-assisted students against a cohort given direct access to the AHA guidelines. Such a design would provide a benchmark for the practical value of AI assistance versus traditional resource retrieval and help quantify the true benefit of the LLM’s synthetic capabilities.

## Conclusions

Our investigation into RAG reveals that while generally beneficial for LLMs accuracy, its impact is conditional, varying with model architecture, scale, baseline capabilities, and retrieved document characteristics. Significantly, open-source models exhibited competitive, and at times superior, performance to proprietary models, even without preprompting, indicating their growing promise for domain-specific applications. RAG did not, however, universally enhance accuracy, occasionally leading to slight performance declines. Moreover, a pilot user study demonstrated that LLM assistance provided no significant accuracy gains for dental students or postgraduates and increased task duration, highlighting practical limitations for current clinical integration. These results necessitate careful consideration in selecting and tailoring LLMs for clinical support and emphasize an urgent need for optimized retrieval methodologies to fully leverage RAG’s potential in advancing dental education and practice. The most important consideration is that, given IE is a life-threatening condition, information obtained from LLMs regarding patient management should always be independently verified before clinical application. The findings of this study indicate that, although LLMs demonstrate potential, their accuracy remains insufficient for unsupervised use in patient care.

## Appendix Supplementary materials

Image, application 1

Image, application 2

**Supplementary Table S1.** Artificial intelligence in dental research checklist for this study. Image, application 3

## Footnotes

Supplementary material associated with this article can be found in the online version at doi:10.1016/j.identj.2025.109344.
