# Supplementary material for: Evaluating Retrieval-Augmented Generation-Large Language Models for Infective Endocarditis Prophylaxis: Clinical Accuracy and Efficiency
Source: Int Dent J. 2025 Dec 25;76(1):109344. doi: 10.1016/j.identj.2025.109344 (PMC12828207; doi:10.1016/j.identj.2025.109344)
Supplement: Supplementary file 1 [file mmc1.docx]

**Supplementary Table S1. Artificial intelligence in dental research checklist for this study.**

| **S. No.** | **Criteria** | | **Fulfillment**  **(Yes/No/Not applicable)** |
| --- | --- | --- | --- |
|  | **Planning and Conducting** | |  |
| 1 | Study Goal | Consider relevance, scope and meaning and limitations of the AI application. | Yes |
| 2 | Study Focus | Clarify if developing a new or validating an existing model, or if scope diagnostics or prognostics etc.. | Yes |
| 3 | Data | Scrutinize the available dataset and mitigate bias, ensuring generalizability. | Yes |
| 4 | Study Aim | Define if the study is exploratory or hypothesis-testing and consider implications for study conception. | Yes |
| 5 | Reference Test | Decide on a justifiable basis for a method to establish a reference test, especially when involving multiple annotators. | Yes |
| 6 | Clustering | Consider clustering of teeth and patients in your dataset, for example during data partitioning (“data snooping bias”). | Not applicable |
| 7 | Test Dataset | Report test metrics from an independent test dataset. | Not applicable |
| 8 | Computational Resources | Consider resources when working with larger datasets or complex models. | Yes |
| 9 | Comparators | Compare your model against relevant comparators (experts, other models) using meaningful metrics. | Yes |
|  | **Reporting** | |  |
| 10 | Title | Define that any kind of AI was used, specify which one and for which focus and problem. | Yes |
| 11 | Abstract | Present a structured summary of the study’s aim, methods, results, and conclusion. | Yes |
| 12 | Introduction | Sum up the clinical background and need of AI solution; achievements and limitations so far; goal of the study; hypothesis (if needed). | Yes |
| 13 | Study design | Assist the reader in understanding your study by providing an overview about the study goal, data characteristics, modeling techniques, evaluation and scope. | Yes |
| 14 | Data | Give details towards the source of data for training and testing, in- and exclusion criteria, sampling framework, fit to target population, heterogeneity, partitioning, and if and where it can be accessed (or why not). | Not applicable |
| 14a | Sampling | Provide inclusion and exclusion criteria, case definition, image type and quality, data source(s)/centers, sampling strategy and information towards heterogeneity. | Not applicable |
| 14b | Data Protection | Provide information how data protection requirements were fulfilled. | Yes |
| 14c | Missing data | Explain how missing data was handled. | Yes |
| 14d | Data Processing | Lay out how data processing (extracted, transposed, loaded, preprocessed) was performed. | Yes |
| 15 | Reference Test | Explain how the reference test was generated, including case definition, grading schemes, test thresholds and unification strategies for multiple labels. | Yes |
| 16 | Sample Size | If your study is hypothesis-testing, provide information how you arrived at your test dataset sample size. | Yes |
| 17 | Model | Provide detail information on model inputs, outputs, intermediate layers, pooling, normalization, regularization, and activation, as well as software packages and hardware used. The structure of the model may be presented. | Yes |
| 17a | Model parameters | Describe how the model parameters were initialized. | Yes |
| 18 | Training | Describe the training procedures including data augmentation techniques, criteria used for stopping the training, hyperparameters and hyperparameter search strategy. For neural networks, at least the learning rate schedule, optimization method, batch size, dropout rates, regularization parameters (if any) and number of epochs should be provided. | Not Applicable |
| 19 | Justify the best- performing model | Describe the method and model metric to select the final model and evaluate it against the hold-out test set. | Not applicable |
| 20 | Evaluation | Describe the primary outcome and outcome metric. Consider further outcomes with relevance to your question. | Yes |
| 21 | Uncertainty | Describe how uncertainties in the model results (comparisons, subgroups) are reflected on. | Yes |
| 22 | Explainability | Lay out how explainability, trustworthiness, and transparency were assessed. | Yes |
| 23 | Results | Provide information on flow of data, including those in- and excluded, and data partitions into training, validation and test dataset. Characterize the dataset. | Not Applicable |
| 23a | Performance Metrics and Data Partitions | The final model’s performance on the test partition should be provided in detail, and benchmarked against current technical standards. Provide uncertainty estimates. Provide information to understand incorrect predictions and explainability. | Yes |
| 24 | Discussion | Provide a summary, a strengths and limitations sections, a section on findings and their implications, and one on future directions. | Yes |
| 25 | Other | Provide information towards authorship and registration, study protocol and potential conflicts of interest. | Yes |

Reference: Schwendicke, F., Singh, T., Lee, J. H., Gaudin, R., Chaurasia, A., Wiegand, T., ... & Krois, J. (2021). Artificial intelligence in dental research: Checklist for authors, reviewers, readers. *Journal of dentistry*, *107*, 103610.
